# Reconstruction of a gene regulatory network of the induced systemic resistance defense response in Arabidopsis using boolean networks

**DOI:** 10.1186/s12859-020-3472-3

**Published:** 2020-04-15

**Authors:** Tania Timmermann, Bernardo González, Gonzalo A. Ruz

**Affiliations:** 1 Laboratorio de Bioingeniería, Facultad de Ingeniería y Ciencias, Universidad Adolfo Ibáñez, Santiago, Chile; 2 Millennium Nucleus Center for Plant Systems and Synthetic Biology, Santiago, Chile; 3 Center of Applied Ecology and Sustainability (CAPES), Santiago, Chile

**Keywords:** Boolean networks, Differential evolution, Gene regulatory networks, Induced systemic resistance, *Paraburkholderia phytofirmans*, *Pseudomonas syringae*

## Abstract

**Background:**

An important process for plant survival is the immune system. The induced systemic resistance (ISR) triggered by beneficial microbes is an important cost-effective defense mechanism by which plants are primed to an eventual pathogen attack. Defense mechanisms such as ISR depend on an accurate and context-specific regulation of gene expression. Interactions between genes and their products give rise to complex circuits known as gene regulatory networks (GRNs). Here, we explore the regulatory mechanism of the ISR defense response triggered by the beneficial bacterium *Paraburkholderia phytofirmans* PsJN in *Arabidopsis thaliana* plants infected with *Pseudomonas syringae* DC3000. To achieve this, a GRN underlying the ISR response was inferred using gene expression time-series data of certain defense-related genes, differential evolution, and threshold Boolean networks.

**Results:**

One thousand threshold Boolean networks were inferred that met the restriction of the desired dynamics. From these networks, a consensus network was obtained that helped to find plausible interactions between the genes. A representative network was selected from the consensus network and biological restrictions were applied to it. The dynamics of the selected network showed that the largest attractor, a limit cycle of length 3, represents the final stage of the defense response (12, 18, and 24 h). Also, the structural robustness of the GRN was studied through the networks’ attractors.

**Conclusions:**

A computational intelligence approach was designed to reconstruct a GRN underlying the ISR defense response in plants using gene expression time-series data of *A. thaliana* colonized by *P. phytofirmans* PsJN and subsequently infected with *P. syringae* DC3000. Using differential evolution, 1000 GRNs from time-series data were successfully inferred. Through the study of the network dynamics of the selected GRN, it can be concluded that it is structurally robust since three mutations were necessary to completely disarm the Boolean trajectory that represents the biological data. The proposed method to reconstruct GRNs is general and can be used to infer other biologically relevant networks to formulate new biological hypotheses.

## Background

### Issues in crop production

Pathogenic microorganisms affecting plant health are a major and chronic threat to sustainable agriculture and ecosystem stability worldwide. In 2004, more than 800 million people did not have adequate food to support an active life and at least 10% of the world food production was lost due to diseased crops [1]. Pest and pathogens are a constant and important threat to crops, which translates into losses of more than 450 billions euros worldwide [2]. On the other hand, the chemical fertilizers and pesticides used in agriculture to increase yields, kill pathogens, pests, and weeds, have a big harmful impact on the ecosystems [3, 4]. Because of current public concerns about the side effects of agrochemicals, there is an increasing interest in improving the understanding of cooperative activities among plants and rhizosphere microbial populations [5]. The use of beneficial bacteria has become a good alternative to solve this problem. Over the last years, they have been used to increase soil fertility, plant growth, and to control phytopathogens as an environmentally sustainable alternative in agriculture [6].

### Use of beneficial bacteria

To use more efficiently formulations of beneficial bacteria as biopesticides, discovering how plant defense works has become more and more important; in particular, the defense response triggered by beneficial microorganisms called induced systemic resistance (ISR). The ISR response can improve plant health by priming the entire plant to increase the defense against various pathogens and insect herbivores [7]. The positive effect of some beneficial bacterial strains on plants infected by bacteria, viruses, fungi, and insects is well reported [8–11], however, the molecular mechanisms and the main signaling pathways involved have not been well characterized yet and also vary from one beneficial bacterial species to another.

### The fate of a cell

Gene expression is a vital task of a cell and an organism as whole to adapt to environmental changes and ensure its survival. Gene regulatory networks (GRNs) coordinates the transcription of genes when is required. A GRN captures dependencies among molecular entities that are part of a system. GRNs are usually represented as graphs where nodes represent molecular entities (i.e., genes, proteins, metabolites) and directed edges represent functional relationships between them (i.e., protein-DNA interactions, protein-protein interactions, microRNA: target interactions, co-expression). The complexity of studying a GRN increases significantly as the number of nodes/genes and connections increases. To tackle this issue several mathematical models have been used. For example, using a mathematical model (Boolean networks) the inference of the GRN underlying the floral transition in plants was successfully achieved [12]. The inferred GRN allowed to point out probable gaps in the biological knowledge of the developmental program and to suggest novel regulatory interactions absent from the starting GRN, built with empirical data without mathematical models.

### GRN inference

The construction of GRNs models from data is typically referred to as a reverse engineering problem [13, 14]. Building GRNs is a difficult task given the large space of possible GRN models that might fit the data and the need to search that space in a reasonable time to derive useful solutions. Several approaches using evolutionary computation (EC) have been proposed to aid in this search.

### Boolean networks

Stuart Kauffman introduced in 1969 [15] a deterministic model called Boolean networks (BNs) which have been widely used to describe and analyze the behavior of a GRN, given a good notion of its qualitative dynamics. The dynamics is represented by the temporal evolution of the gene or protein states. For this reason, they are a good mathematical model for research where little or insufficient knowledge exists (molecular data, especially quantitative data), allowing to carry out exploratory studies in processes scarcely studied in model organisms, or even in processes well studied in model organisms, but not studied in other species. In a BN, the nodes represent genes or proteins, which can either be active (value 1) or inactive (value 0) and the edges represent regulatory relations amongst the genes. Each node updates its value accordingly to a local Boolean function that depends only on the values of the parent nodes of the node been updated, and an updating scheme (synchronous, asynchronous, block-sequential, etc.). For a network with *n* nodes, there are 2^*n*^ possible configurations (or states), therefore, any updates of the network will remain within the possible configurations. The network has two types of steady states or attractors. A state that remains the same after the network is updated is known as a fixed point, whereas a sequence of state vectors that loop is known as a limit cycle.

### Three approaches to reconstruction

There are three common approaches for BN reconstruction: (1) based on transcriptional time-series data of wild-type organisms [16], (2) based on transcriptional analysis of a set of knockouts or mutants [17], or (3) prior knowledge of the process that wants to be modeled (regulatory relations identified in previous works) [18]. When the data for inference comes from empirical data, the construction of the topology of a BN involves two key steps: first, the experimental data (protein concentration or gene expression) must be discretized into binary values and second, the binary profiles are used to build the BN that best captures the Boolean trajectories.

### EC algorithms

The use of computational intelligence becomes very valuable for the reconstruction of BNs. Especially when it comes to network reconstruction with topological and (or) dynamical constraint. EC algorithms are a family of population-based trial and error problem solvers with a metaheuristic or stochastic optimization character. Following this line of research, the Bees Algorithm has been used to infer BNs with predefined attractors [19] as well as networks for biotechnological applications [20]. Additionally, a genetic algorithm was used to find the network’s parameters from time-series data of gene expression, to infer a GRN related to salt stress response in *Arabidopsis thaliana* [21]. Finally, the study performed by [12] uses genetic programming to construct Boolean networks representing the GRN that controls the shoot apical meristem during the floral transition in plants. These BNs were constructed using a defined network topology based on gene expression data from *in situ* hybridization. The inferred GRN complements the original topology with new regulatory edges confirmed by independent laboratory work [12].

### Wildtype networks

In cases where there exists a base network model, sometimes called the *wildtype* network, several inference frameworks have being proposed to generate alternative network structures that share the same functionality (for example, a set of specific attractors) as the wildtype. An inference framework is proposed that uses Markov Chain Monte Carlo to sample the network space to search for BNs with 10 predefined attractors [22]. Another work develops a set of procedures to predict putative missing interactions in the *A. thaliana* root stem cell niche network model [23]. The set of procedures consisted of adding all the possible missing interactions one-by-one to the model without contradicting the experimental data and measuring the effect of these additional interactions in the set of the attractors. The search of the network space with functionally equivalent networks is also known as the *neutral space*.

### Neutral space

An example of a neutral space analysis appears in [24], using the *Schizosaccharomyces pombe* (fission yeast) cell-cycle network as the wildtype network and an evolution strategy to find functionally equivalent BNs that had the same cell-cycle dynamics as the wildtype network. While all of these inference frameworks have shown to be effective, all of them require as the starting point an existing BN (wildtype) and its respective dynamics, and therefore, its attractors. In this work, we do not have a wildtype network or base model to use for inference, therefore we can not use the methodologies described in the previous works.

### *Paraburkholderia phytofirmans*

We recently described that the beneficial bacterium *Paraburkholderia phytofirmans* PsJN triggers ISR in *A. thaliana* plants, protecting them from the bacterial pathogen *Pseudomonas syringae* DC3000 [25]. Additionally, in a recent study, the temporal changes in the transcriptome of PsJN-inoculated plants before and after *Pst* DC3000 infection was revealed [26]. To get a better understanding of the fine-tuning regulation which helps explain this ISR phenomenon at a molecular level, we inferred an underlying GRN with eight key genes. For this, we used an EC approach, specifically, differential evolution (DE), and gene expression time-series data of eight specific genes known to play a role in plant responses to biotic stress.

### Genes in the defense backbone

We used *WRKY70*, *WRKY54*, *WRKY33*, *PR1*, *ERF1*, *MYC2*, *PDF1.2* and *LOX2*, which are key genes and transcription factors for the hormonal modulation of plant immunity, involving the cross-talk between salicylic acid (SA), jasmonic acid (JA), and ethylene (ET) signaling pathways [27–29]. These genes have been well studied over the years, mainly in the model plant *A. thaliana*. For this reason, these genes should be considered part of the backbone of any GRN involved in a defense response such as ISR. Additionally, most of the genes of this group have shown significant changes in their expression in Arabidopsis plants inoculated with strain PsJN and infected with *P. syringae* DC3000 in comparison to non-inoculated plants [25].

With this transcriptional data, we generated a threshold Boolean regulatory network that underlies this defense response sparsely studied at the molecular level, with the objective to predict novel gene regulatory relationships that may help to formulate further hypotheses for future research.

## Results and discussion

### Hormonal crosstalk in plant defense

Phytohormone crosstalk is crucial for plant defense against pathogens and insects, in which SA, JA, and ET hormones play key roles [27, 30]. Therefore, the expression of eight defense-related genes of *A. thaliana*, representing the SA, JA and ET hormonal pathways, was analyzed. The *PATHOGENESIS-RELATED GENE 1* (*PR1*; AT2G14610) belonging to a group of genes that code for proteins with antimicrobial activity [31], is the best-characterized and most common marker for the SA-signaling pathway [32] and its expression is induced in response to a variety of pathogens. The transcription factors WRKY54 (AT2G40750) and WRKY70 (AT3G56400) are induced by SA and can regulate the expression of SA-responsive genes, such as *PR1* [33, 34]. The transcription factor WRKY33 (AT2G38470) has a role as a negative regulator of SA-dependent defense responses and appears to directly control the expression of *ORA59* during the later stages of pathogen infection [35, 36]. The transcription factor MYC2 (AT1G32640) differentially regulates two different classes of JA-responsive genes: MYC2 functions as a positive regulator of JA-responsive genes such as *VSP2* and *LOX2* (MYC branch); whereas it acts as a negative regulator of JA/ET-responsive genes, such as *PDF1.2*, which are activated by the transcription factors ETHYLENE RESPONSE FACTOR1 (ERF1; AT3G23240) and ORA59 (ERF branch) [37–40]. *LIPOXYGENASE2* (*LOX2*; AT3G45140) encodes a key enzyme in the octadecanoid pathway leading to JA biosynthesis [41] and the *PLANT DEFENSIN 1.2*(*PDF1.2*; AT5G44420) encodes a plant defensin with antimicrobial properties [42].

### Time-series data

**Fig. 1:**
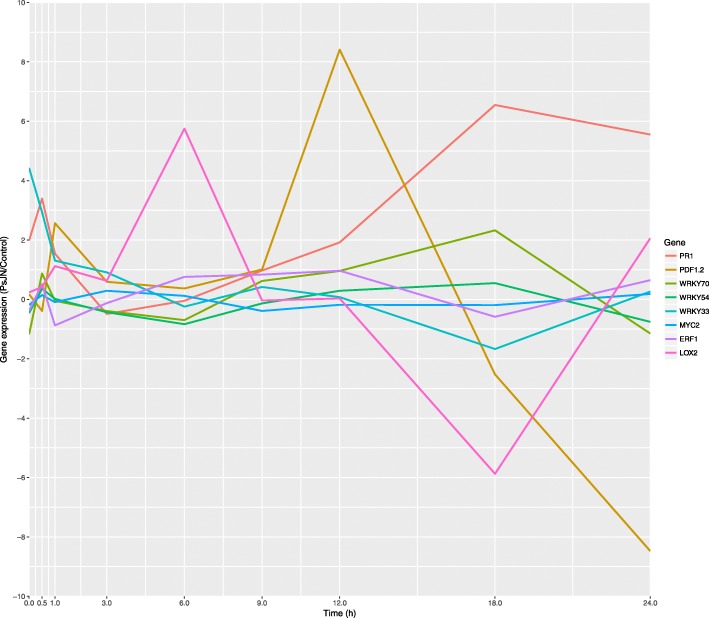
Expression patterns of *Arabidopsis thaliana* defense-related genes in response to *Paraburkholderia phytofirmans* PsJN before the infection with *Pseudomonas syringae* DC3000 (0 h) and after 0.5, 1, 3, 6, 9, 12, 18 and 24 h of the infection. Data are means of three to five biological replicates per treatment, each considering tissue from four plants and two technical replicates. Normalization was performed with the housekeeping *SAND* family gene (AT2G28390)

For each time point, the expression values in the strain PsJN-inoculated plants were normalized with respect to the expression values of the non-inoculated plants (control). Then, if a gene shows an expression greater than zero, it means that strain PsJN is activating it. On the contrary, if a gene shows an expression below zero, it means that strain PsJN is repressing it. The expression patterns of *PR1*, *PDF1.2*, *WRKY70*, *WRKY54*, *WRKY33*, *MYC2*, *ERF1*, and *LOX2* genes are shown in Fig. 1.

These expression values were binarized following the rules explained in the “Methods” section and the resulting matrix is shown in Table 1. This matrix was the input and the objective function for the DE used to infer the GRN that would explain the ISR response triggered by *P. phytofirmans* PsJN in *A. thaliana* plants infected with the phytopathogenic strain *Pst* DC3000.

**Table 1:** Temporal evolution of the state vectors of the eight genes used to reconstruct gene regulatory networks using differential evolution

Time(h)	PR1	PDF1.2	WRKY70	WRKY54	WRKY33	MYC2	ERF1	LOX2
0	1	1	0	0	1	0	0	1
0.5	1	0	1	1	1	1	1	1
1	1	1	0	1	1	0	0	1
3	0	1	0	0	1	1	0	1
6	0	1	0	0	0	1	1	1
9	1	1	1	0	1	0	1	0
12	1	1	1	1	1	0	1	1
18	1	0	1	1	0	0	0	0
24	1	0	0	0	1	1	1	1

### Networks found by DE

**Fig. 2:**
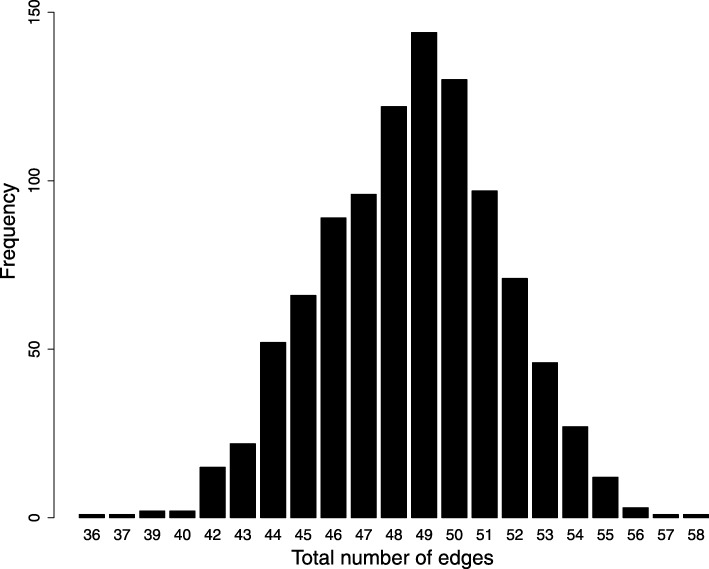
Frequency distribution of the total number of edges of the resulting 1000 threshold Boolean networks. The networks were found by differential evolution that contained the desired Boolean trajectory

**Fig. 3:**
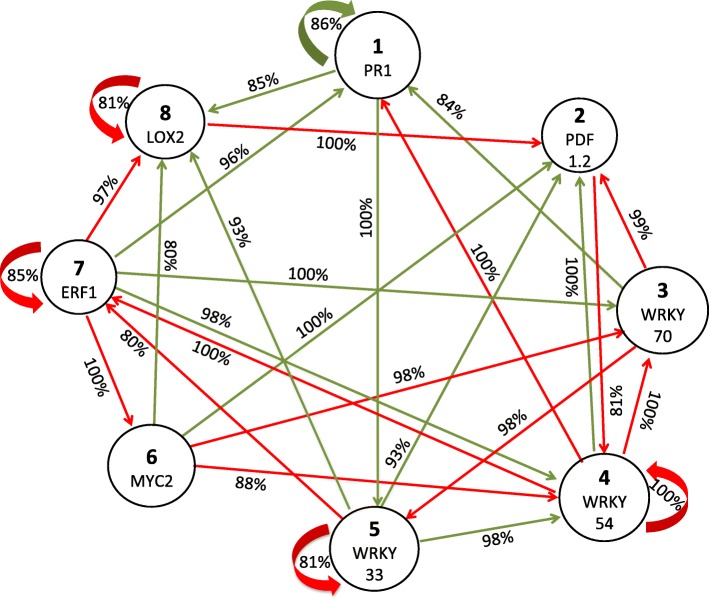
Consensus network constructed with 1000 threshold Boolean networks. The percentage represents how many times the edge appeared within the 1000 inferred networks. The consensus network shows only the edges that appear in more than 80% of the inferred networks. The green edges represent positive weights (activations, +1) and the red edges represent negative weights (inhibitions, -1)

Using computer simulation, 1000 solutions (networks) were found. The distribution of the number of edges of the 1000 networks is shown in Fig. 2. We found that the most frequent number of edges is 49, the maximum number of edges found for a network is 58, and the minimum number of edges is 36. Given that many networks satisfy the reconstruction restrictions (temporal evolution of state vectors), a consensus network approach was used to identify regulations among the genes that are more plausible (Fig. 3). The cutting threshold for the edges used in the consensus network was 80%, which means that when the edge appeared in more than 80% of the inferred networks, it was considered for the consensus network. Additionally, the labels of the edges (positive or negative) represent the percentage of how many times the edge appeared within the 1000 inferred networks.

### Dynamics of a candidate solution: network 707

**Fig. 4:**
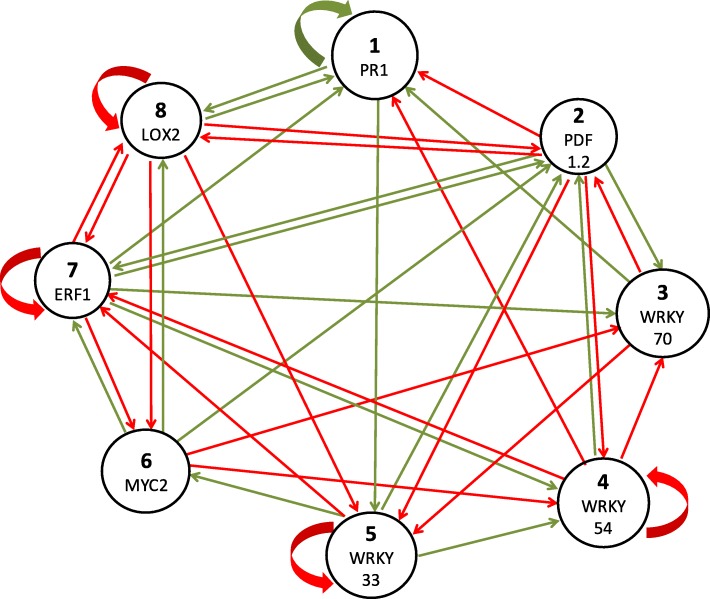
Network N^**o**^ 707 is the most similar Gene Regulatory Network solution to the consensus network. The threshold values for each node are *Θ*= (1; 1; 2; 0; -2; 0; -2; -1). The green edges represent positive weights (activations, +1) and the red edges represent negative weights (inhibitions, -1)

To study the dynamics of a GRN represented as a BN, a single network was selected within the 1000 inferred networks (Fig. 4). The network N^**o**^ 707, henceforth 707, was the most similar to the consensus network in terms of the topology. Therefore, this network was used to study the global dynamical behavior of a GRN involved in the ISR response in plants. The BN considered has eight nodes therefore there are 256 (2^8^) possible configurations. Given the deterministic nature of this model, the network converges to steady states, also known as attractors (fixed points and limit cycles).

**Fig. 5:**
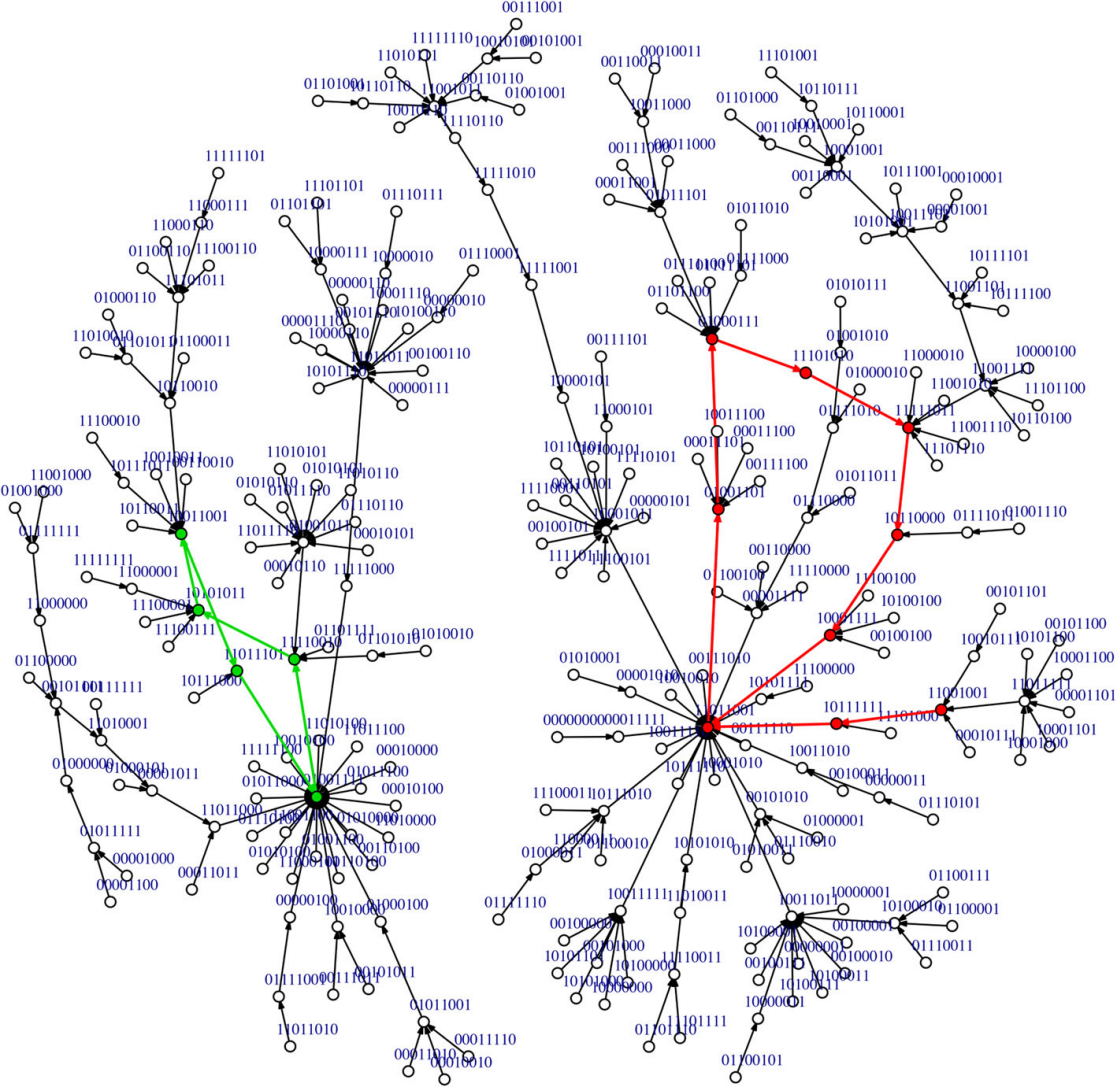
State transition graph of the network N^**o**^ 707 using the parallel updating scheme. The attractors are two limit cycles. One limit cycle (red path) represents the temporal evolution of the state vectors from time 1 h to time 24 h. The other limit cycle is denoted with a green path

After a parallel updating scheme, the dynamics of the network 707 converged to two limit cycles, one of length 7 and other of length 5, red and green paths, respectively, in Fig. 5. The limit cycle, which attracted 60% of the states, (red path; basin size: 155) represents the plant status one hour (1 h) after the infection with the pathogen until 24 hours (24 h) after the infection, being part of the limit cycle all other times that are contemplated in this time frame (3, 6, 9, 12 and 18 h). The other limit cycle has a basin of attraction of 101 states and it does not represent any of the plant states studied in this work.

### Pruning network 707

**Fig. 6:**
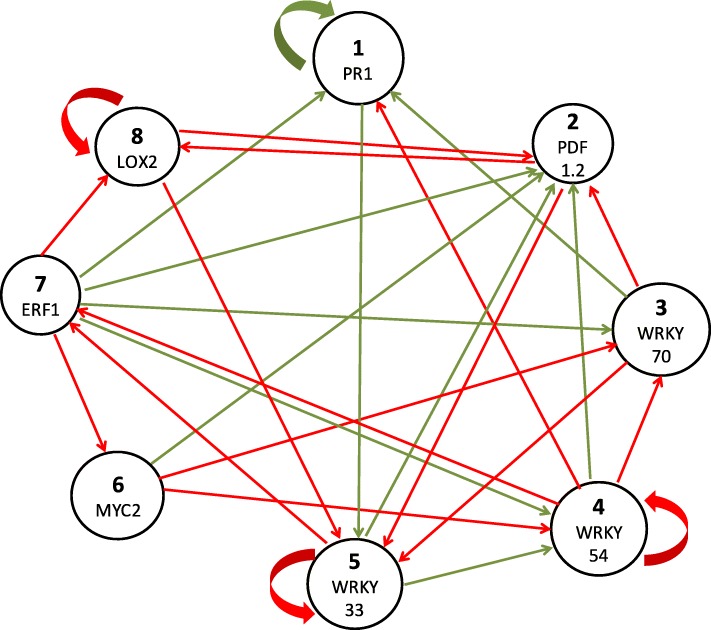
Network N^**o**^ 707 with biological restrictions. The threshold values for each node are *Θ*= (2; 0; 0; 2; -2; 0; -1; -2). The green edges represent positive weights (activations, +1) and the red edges represent negative weights (inhibitions, -1)

Because the inference of BNs from time-series data of gene expression is a mathematical problem and not a biological one, the set of networks that satisfy the objective function (desired Boolean trajectory of Table 1), in some cases may be very extensive. Many of these networks have connections between genes (edges) that have no biological meaning. Therefore, to obtain a more reliable GRN, biological restrictions were applied to the topology of the network 707. Twelve regulations (edges) were removed based on empirical data reported in the literature, for example, the positive edge from *MYC2* (node 6) to *ERF1* (node 7) is incorrect since MYC2 and ERF1 are antagonistic transcription factors. This new network was called 707-BR (Fig. 6) and is composed of 28 edges instead of 40 that had the original network 707.

**Fig. 7:**
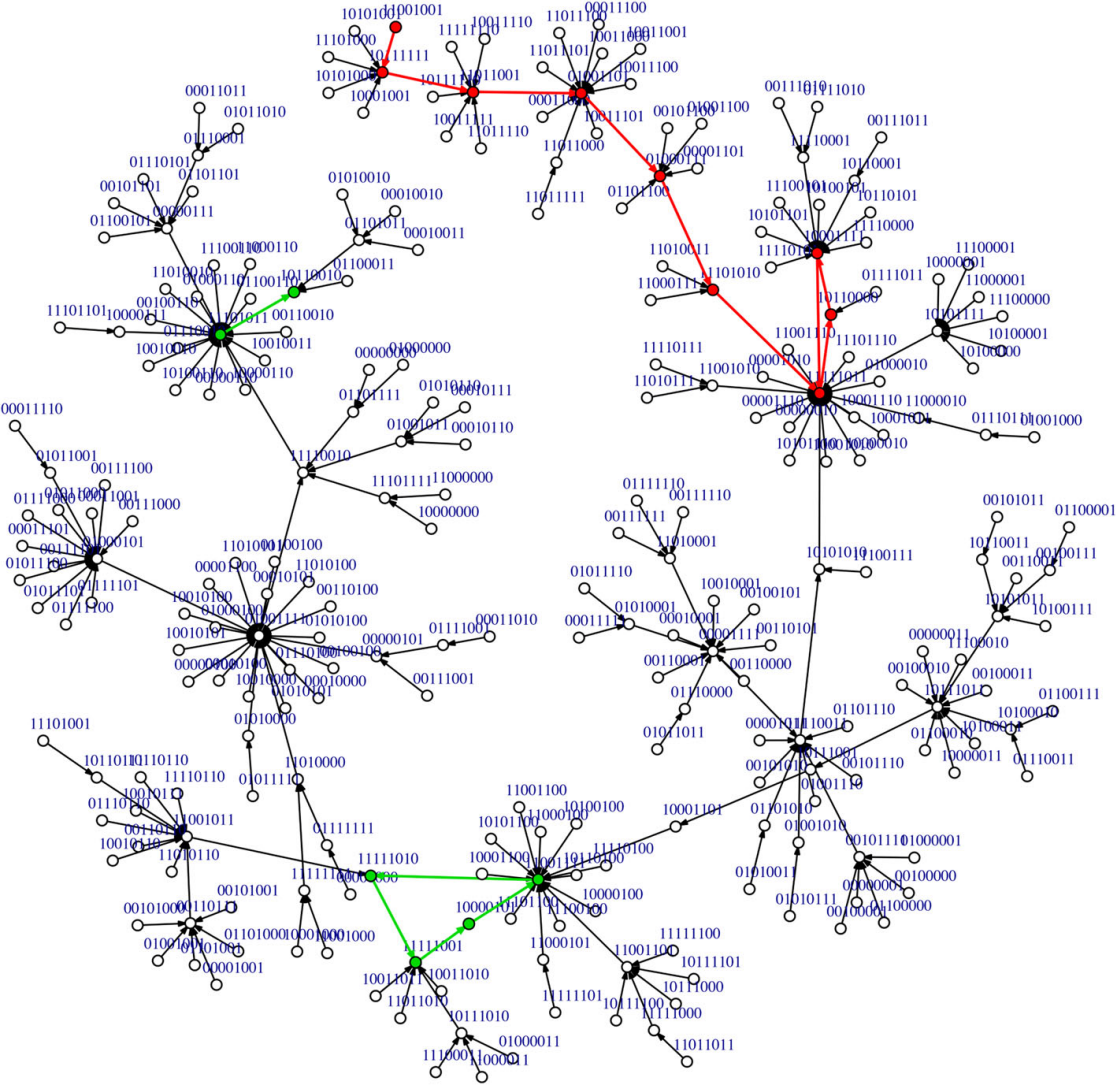
State transition graph of the network N^**o**^ 707 with biological restrictions (707-BR) using the parallel updating scheme. The attractors are three limit cycles. One limit cycle (red path) represents the temporal evolution of sate vectors from time 12 h to time 24 h. The other limit cycles are denoted with a green path

After a parallel updating scheme, the dynamics of the network 707-BR converged to three limit cycles of length 4, 3 and 2 (Fig. 7). The limit cycle of length 3 that attracted most states (red path in Fig. 7; basin size: 104) represents the plant status 12,18, and 24 h after the infection with the pathogen. The Boolean trajectory represented in Table 1 was attracted by this limit cycle, where once it falls inside, it continues to cycle continuously between the 12, 18 and 24 h states. The limit cycle of length 4 has a basin of attraction of 67 states (green path in Fig. 7) and the limit cycle of length 2 has a basin of attraction of 85 states. Both of them do not represent any of the plant states studied in this work.

### Structural robustness of network 707-BR

#### Single mutation

To study the structural robustness of the network 707-BR, topological changes with biological support were carried out. These changes represented genetic mutations in fundamental genes to achieve, through the SA, JA or ET signaling pathways, a successful ISR response triggered by strain PsJN, as was previously described in [25]. In the first network, henceforth 707-BR.M1, the transcription factor WRKY70 (node 3) was removed, this factor is directly involved in the SA signaling pathway activated by strain PsJN [43]. This topological change represents a single mutant Arabidopsis plant, i.e. the mutant line *wrky70-1*, where the SA-induced expression of *WRKY70* is completely blocked. A direct consequence of this mutation is the non-activation of the defense gene *PR1*. To study the robustness of the network 707-BR.M1 a parallel updating scheme was carried out. The result of the dynamics was a single attractor as a limit cycle of length 5. Interestingly, the dynamics follows the same trajectory as the wild type network (707-BR) until the vector that represents the time point 12 h. The vector of this point and of the time point 18 h changed, activating the JA/ET responsive-defense genes *WRKY33* (node 5) and *PDF1.2* (node 2), which is consistent with the biological process since the hormone signaling pathways SA and JA/ET are mainly antagonistic [27, 44].

#### Double mutation

To deepen the study of the structural robustness of the network, an additional mutation to the network 707-BR.M1 was made. Then, the new network called 707-BR.M2 represented a double mutant Arabidopsis plant impaired in SA and ET signaling pathways. The removed nodes were *WRKY70* (node 3) and *ERF1* (node 7). After a parallel updating scheme the dynamics showed two attractors, a fixed point (1 1 1 0 1 1 1 1) and a limit cycle of length 2. Concerning the trajectory of the network 707-BR.M1 dynamics, this double mutant added two changes that took it out of the initial trajectory, one in time 0.5 h where the genes *PR1* and *WRKY54* were inactivated, and on the contrary, *MYC2* gene was activated. These results are in agreement with empirical data of the SA, JA and ET crosstalk where the absence of the transcription factor WRKY70 does not allow the activation of *PR1* [44]. On the other hand, as *MYC2* is a negative regulator of *ERF1* [45] the absence of the node ERF1 leads to an activation of *MYC2*. Even with these changes, the Boolean trajectory can return to the trajectory of the network 707-BR.M1, evidencing intrinsic robustness of the original network (wild type network 707-BR).

#### Triple mutation

To challenge the robustness of the GRN, a triple mutant was carried out. The new network called 707-BR.M3 has no incidence of nodes 3, 6 and 7, which represents *WRKY70*, *MYC2* and *ERF1* genes, respectively. In other words, this network is impaired in SA, JA, and ET signaling pathways. As expected, the Boolean trajectory of the triple mutant network is practically 100% different than the Boolean trajectory of the wild type network 707-BR. The dynamics has only one attractor, a limit cycle of length 2. This result was expected because defense systems in plants need at least one integral hormonal pathway to activate defense-related genes. Additionally, these mathematical results are in agreement with the biological response of a triple mutant plant impaired in SA, JA and ET signaling pathways infected with *Pst* DC3000, where strain PsJN was unable to protect this mutant line [25].

### On the use of DE for GRN inference

The use of experimental data usually leads to work with noise in the data. For this reason, the inference of GRNs can become a difficult task. With the time-series data of gene expression developed for this work, the classical algorithms to infer GRNs, REVEAL [13] and Best Fit Extension [46] were unable to infer GRNs without error. Because of this, an EC algorithm, the genetic algorithm previously used to infer GRNs [21], was used. However, it was not possible to infer GRNs with zero error. On the contrary, when the DE algorithm was applied, the inference of GRNs with zero error was achieved. The use of prior knowledge of the biological process under study is important when defining biological constraints to infer networks. With this in mind, the reconstruction of a GRN can be as similar as possible to the cellular reality.

## Conclusions

A computational intelligence approach is presented to reconstruct a GRN underlying the ISR defense response in plants using gene expression time-series data of *A. thaliana* colonized by *P. phytofirmans* PsJN and subsequently infected with *P. syringae* DC3000. With the DE, 1000 GRNs from time-series data were successfully inferred. Through the study of the network’s dynamics of the selected GRN (707-BR), we showed that this network is structurally robust since three mutations were necessary to completely disarm the Boolean trajectory that represents the biological data. Also, this is in agreement with the biological process under study, the immune system.

With the use of BN models, it is possible to make qualitative predictions and formulate new biological hypotheses. The combination of predictive modeling with systematic experimental verification will be required to gain a deeper insight into biological processes. For example, by knowing the GRN that coordinates the defense response mediated by a common beneficial bacterium, it will be possible to model and predict plant responses to exogenous and endogenous changes that involve genes from the studied network, allowing a more effective design of the use of beneficial bacteria to improve the yield of important crops.

## Methods

### Time-series data from gene expression

#### Infection assay

To construct the time-series data from gene expression an infection assay with *P. syringae* (*Pst*) DC3000 (provided by the Faculty of Biological Sciences, Pontificia Universidad Católica de Chile) in Arabidopsis plants (Arabidopsis seeds, Columbia 0 ecotype, from the Arabidopsis Biological Resource Center (ABRC)) was carried out. Sterilized *A. thaliana* seeds (Col-0) were sown in square Petri dishes with solid 50% Murashige & Skoog medium (0.8% agar). Half of the Petri dishes were inoculated with *P. phytofirmans* PsJN and the other half were not inoculated (control plants). Plates were located vertically in a growth chamber at 22 ^∘^C with a photoperiod of 16/8 h (light/dark). King’s B medium supplemented with 50 *μ*g/ml of rifampicin and 50 *μ*g/ml of kanamycin as selection antibiotics was used to grow the virulent strain of the phytopathogenic bacterium *P. syringae pv. tomato* DC3000, henceforth *Pst* DC3000. Thirteen days after sowing (13 DAS) *A. thaliana* plants with four visible leaves (LP.04 stage [47]) were sampled and stored in 1.5 ml Eppendorf tubes containing RNAlater^TM^ (Ambion, Austin, TX, U.S.A.) according to the manufacturer’s instructions. Treatment “0 h” in the qRT-PCR results corresponds to this group of plants. After strain *Pst* DC3000 infection, four randomly selected *Pst*-infected plants were sampled and stored in 1.5 ml Eppendorf tubes containing RNAlater^TM^ (Ambion), according to the manufacturer’s instructions. The sampling times after infection were 0.5, 1, 3, 6, 9, 12, 18 and 24 h. Five pools with four plants each were collected per treatment (non-inoculated plants/*Pst* and strain PsJN-inoculated plants/*Pst*).

#### RNA extraction and cDNA synthesis

For RNA extraction and cDNA synthesis, the same methodology described in [25] was used.

#### Gene expression measurement

To quantify the gene expression a real-time RT-PCR was performed following the same methodology described in [25]. Also, Supplementary Table S1 gives the sequences of all primer pairs and their references (if applicable).

#### Data binarization

To visualize the effect of strain PsJN in a graph, for each time, the expression of the non-inoculated plants (control) was subtracted to the expression of strain PsJN-treated plants. Finally, these expression data were binarized according to the following rule: negative expression values (genes down-regulated by strain PsJN) took value “0” and positive expression values (genes up-regulated by strain PsJN) took value “1”. Table 1 shows the binarized expression data that represents the desired Boolean trajectory, which in turn is the function that the inferred network must satisfy.

### Network reconstruction using DE

To infer BN an EC approach was used. In particular, it was considered a threshold BN with *n* nodes, where edges have weights and each node has a threshold value, then each node *x*_*i*_ (for $i=1,\dots,n$) updates its value by a Heaviside function in the following way:

1 $$\begin{array}{@{}rcl@{}} x_{i}(t+1)&= H\Bigg(\sum_{j=1}^{n} \omega_{ij}x_{j}(t)- \theta_{i}\Bigg) \\&= \left\{ \begin{array}{ccccc} 0 &\quad if \quad&\sum_{j=1}^{n} \omega_{ij}x_{j}(t) - \theta_{i} < 0 \\  1 &\quad if \quad&\sum_{j=1}^{n} \omega_{ij}x_{j}(t) - \theta_{i} \geq 0 \end{array} \right. \end{array} $$

where *ω*_*ij*_ is the weight of the edge coming from node *j* into node *i*, and *θ*_*i*_ is the activation threshold of node *i*. The set of weights and thresholds of the network are the parameters that must be inferred given a desired Boolean trajectory that the network must satisfy. For this, DE was used [48], where the weight matrix and threshold vector of a threshold BN is represented by a vector, which is built by concatenating the weight matrix’s rows and the threshold vector. Starting from random values, candidate solutions (vectors) are evolved through DE until the bit hit error between the desired Boolean trajectory and the Boolean trajectory generated by the network is zero. The simulations were carried out using the open-source R software environment for statistical computing running on a 2.8 GHz Intel Core i7 and 8 GB-RAM computer. In particular, for DE, we used the function DEoptim from [49]. A population of size 1000 was used, with a maximum iteration of 5000, and the search range for the network values (weights and thresholds) was set to the real interval [-2; 2]. Default values were considered for the rest of the user-defined parameters for DE (information can be found using the help function in R for the DEoptim function). The consensus network was built with 1000 solutions (networks).

## Supplementary information

**Additional file 1** Supplementary file 1: Table S1. List of real time RT-PCR primer pairs. Melting temperature and references are indicated.

## Data Availability

All the necessary data used in this study has been provided in the manuscript and the Supplementary files. The software used to infer networks is open source/freely available and has been cited in the study.

## Abbreviations

ISR: Induced systemic resistance
GRN: Gene regulatory network
EC: Evolutionary computation
BN: Boolean network
RNA: Ribonucleic acid
PCR: Polymerase chain reaction
GOI: Gene of interest
HK: Housekeeping
DE: Differential evolution
SA: Salicylic acid
JA: Jasmonic acid
ET: Ethylene
