# Supplementary material for: Reconstruction of a gene regulatory network of the induced systemic resistance defense response in Arabidopsis using boolean networks
Source: BMC Bioinformatics. 2020 Apr 15;21:142. doi: 10.1186/s12859-020-3472-3 (PMC7157984; doi:10.1186/s12859-020-3472-3)
Supplement: Supplementary file 1 — Additional file 1 Supplementary file 1: Table S1. List of real time RT-PCR primer pairs. Melting temperature and references are indicated. [file 12859_2020_3472_MOESM1_ESM.pdf]

Supplementary Material

Supplementary Table S1. List of real time RT-PCR primer pairs. Melting temperature and references are indicated.

Locus (Name)	Primer pairs (5′-3′)	Tm (°C)	Reference
AT2G14610 (*PR1*)	F: GTGGGTTAGCGAGAAGGCTA<br>R: ACTTTGGCACATCCGAGTCT	59	[1]
AT5G44420 (*PDF1.2*)	F: CTTGTTCTCTTTGCTGCTTTCGAC<br>R: ATGCATTACTGTTTCCGCAAACC	61	[2,3]
AT3G56400 (*WRKY70*)	F: CGTCATCATGGTTCGTCCA<br>R: CCACCTCCAAACACCATGAGAT	60	[4]
AT2G40750 (*WRKY54*)	F: CGGAAAAGACATTGGCTGCT<br>R: CCTGCGTCTATTGCTGTCAC	60	Designed in this study
AT2G38470 (*WRKY33*)	F: CTTGACGACGGTTACAGATG<br>R: GTGTTTCCCTTCGTAGGTTG	60	[5]
AT1G32640 (*MYC2*)	F: GATGAGGAGGTGACGGATACGGAA<br>R: CGCTTTACCAGCTAATCCCGCA	62	[6,7]
AT3G23240 (*ERF1*)	F: TCGGCGATTCTCAATTTTTC<br>R: ACAACCGGAGAACAACCATC	58	[8]
AT3G45140 (*LOX2*)	F: ATCAACGCTCGTGCACGCCA<br>R: CCGCGGGTAAGCCTTCCTGG	62	[3]
(AT2G28390) (*SAND*)	F: AACTCTATGCAGCATTTGATCCACT<br>R: TGATTGCATATCTTTATCGCCATC	59	[9]

References of the Supplementary Material

1. Armijo, G., Salinas, P., Monteoliva, M. I., Seguel, A., García, C., Villarroel-candia, E., Song, W., Krol, A. R. Van Der, Álvarez, M. E., Holuigue, L.: A salicylic acid – induced lectin-like protein plays a positive role in the effector-triggered immunity response of *Arabidopsis thaliana* to *Pseudomonas syringae* Avr-Rpm1. Mol. Plant-Microbe Interact. **26**, 1395–1406 (2013)
2. Cartieaux, F., Contesto, C., Gallou, A., Desbrosses, G., Kopka, J., Taconnat, L., Renou, J., Touraine, B.: Simultaneous interaction of *Arabidopsis thaliana* with *Bradyrhizobium sp*. strain ORS278 and *Pseudomonas syringae* pv. *tomato* DC3000 leads to complex transcriptome changes. Mol. Plant-Microbe Interact. **21**, 244–259 (2008)
3. Poupin, M. J., Timmermann, T., Vega, A., Zúñiga, A., González, B.: Effects of the plant growth-promoting bacterium *Burkholderia phytofirmans* PsJN throughout the life cycle of *Arabidopsis thaliana*. PLoS One. **8**, 69435 (2013)
4. Jiang, C. H., Huang, Z. Y., Xie, P., Gu, C., Li, K., Wang, D. C., Yu, Y. Y., Fan, Z. H., Wang, C. J., Wang, Y. P., Guo, Y. H., Guo, J. H.: Transcription factors WRKY70 and WRKY11 served as regulators in rhizobacterium *Bacillus cereus* AR156-induced systemic resistance to *Pseudomonas syringae* pv. *tomato* DC3000 in Arabidopsis. J Exp Bot. **67**, 157-174 (2015)

5. Wang, C., Ding, Y., Yao, J., Zhang, Y., Sun, Y., Colee, J., Mou, Z.: Arabidopsis Elongator subunit 2 positively contributes to resistance to the necrotrophic fungal pathogens *Botrytis cinerea* and *Alternaria brassicicola*. Plant J., **83**, 1019-1033. (2015)
6. Czechowski, T., Bari, R., Stitt, M., Scheible, W.-R., Udvardi, M.: Real-time RT-PCR profiling of over 1400 Arabidopsis transcription factors: Unprecedented sensitivity reveals novel root-and shoot-specific genes. Plant J. **38**, 366–379 (2004)
7. Pozo, M., Van Der Ent, S., Van Loon, L. C., Pieterse, C.: Transcription factor MYC2 is involved in priming for enhanced defense during rhizobacteria-induced systemic resistance in *Arabidopsis thaliana*. New Phytol. **180**, 511–523 (2008)
8. Millet, Y., Danna, C., Clay, N., Songnuan, W., Simon, M., Werck-Reichhart, D., Ausubel, F.: Innate immune responses activated in Arabidopsis roots by microbe-associated molecular patterns. Plant Cell. **22**, 973–990 (2010)
9. Czechowski, T., Stitt, M., Altmann, T., Udvardi, M.K., Scheible, W.-R.: Genome-wide identification and testing of superior reference genes for transcript normalization in arabidopsis. Plant Physiol. **139**, 5–17 (2005)
